# p53 in the Molecular Circuitry of Bone Marrow Failure Syndromes

**DOI:** 10.3390/ijms241914940

**Published:** 2023-10-06

**Authors:** Jeanne Rakotopare, Franck Toledo

**Affiliations:** 1 Genetics of Tumor Suppression, Institut Curie, CEDEX 05, 75248 Paris, France; jeanne.rakotopare@curie.fr; 2 CNRS UMR3244, 75005 Paris, France; 3 Faculty of Science and Engineering, Sorbonne University, 75005 Paris, France; 4 Institut Curie, PSL Research University, 75005 Paris, France

**Keywords:** p53, DREAM repressor complex, dyskeratosis congenita, Fanconi anemia, Diamond–Blackfan anemia, microcephaly, cerebellar hypoplasia

## Abstract

Mice with a constitutive increase in p53 activity exhibited features of dyskeratosis congenita (DC), a bone marrow failure syndrome (BMFS) caused by defective telomere maintenance. Further studies confirmed, in humans and mice, that germline mutations affecting *TP53* or its regulator *MDM4* may cause short telomeres and alter hematopoiesis, but also revealed features of Diamond–Blackfan anemia (DBA) or Fanconi anemia (FA), two BMFSs, respectively, caused by defects in ribosomal function or DNA repair. p53 downregulates several genes mutated in DC, either by binding to promoter sequences (*DKC1*) or indirectly via the DREAM repressor complex (*RTEL1*, *DCLRE1B*), and the p53-DREAM pathway represses 22 additional telomere-related genes. Interestingly, mutations in any DC-causal gene will cause telomere dysfunction and subsequent p53 activation to further promote the repression of p53-DREAM targets. Similarly, ribosomal dysfunction and DNA lesions cause p53 activation, and p53-DREAM targets include the DBA-causal gene *TSR2*, at least 9 FA-causal genes, and 38 other genes affecting ribosomes or the FA pathway. Furthermore, patients with BMFSs may exhibit brain abnormalities, and p53-DREAM represses 16 genes mutated in microcephaly or cerebellar hypoplasia. In sum, positive feedback loops and the repertoire of p53-DREAM targets likely contribute to partial phenotypic overlaps between BMFSs of distinct molecular origins.

## 1. Too Much of a Good Thing: The p53^Δ31^ Mutant Mouse Models a Telomere Biology Disorder

*TP53*, the gene encoding tumor suppressor p53, is the most frequently mutated gene in human sporadic tumors [1], and Li–Fraumeni syndrome in cancer predisposition is caused by the germline inactivating *TP53* mutations [2]. Most *TP53* mutations in cancers affect the core DNA-binding domain of the protein and alter its capacity to bind specific DNA sequences within target gene promoters [3]. However, the p53 protein contains a second DNA-binding domain in its carboxyl terminus [4], and post-translational modifications of this C-terminal domain were proposed to regulate p53 stability and activity [5]. To determine the biological importance of the p53 C-terminal domain, we targeted a nonsense mutation at the mouse *Trp53* locus, which created an allele encoding p53^Δ31^, a mutant p53 that lacks the last 31 amino acid residues [6]. In p53^Δ31/Δ31^ mouse embryonic fibroblasts (MEFs), the p53^Δ31^ protein appeared more stable than wild-type (WT) p53 and correlated with increased activity, as exemplified by a stronger transactivation of p53 target genes (e.g., *Cdkn1a*, *Mdm2*), a more efficient cell cycle arrest response to γ-irradiation, and accelerated senescence [6]. Furthermore, thymocytes are classically used to evaluate radiation-induced apoptosis in mice [7], and an increased apoptotic response was observed in irradiated p53^Δ31/Δ31^ thymocytes [6]. These results clearly established that a loss of the p53 C-terminal domain led to increased p53 activity. The same conclusion was reached with the p53^ΔCTD^ mutant mouse, another model characterized by a smaller deletion of 24 C-terminal residues [8].

Importantly, following intercrosses of p53^+/Δ31^ mice, p53^Δ31/Δ31^ mice were born in Mendelian proportions, but they were much smaller than their WT or p53^+/Δ31^ littermates, exhibited darker footpads and tails, and most died before weaning. Upon dissection, p53^Δ31/Δ31^ mice exhibited cerebellar hypoplasia, hypogonadism in males, and hypertrophic hearts typical of anemic animals. Accordingly, sternum sections from p53^Δ31/Δ31^ mice revealed bone marrow hypocellularity, hematopoietic progenitors were very scarce, and hemograms revealed severe pancytopenia, establishing bone marrow failure as a likely cause of premature death [6]. Similar phenotypic traits had been reported in several mouse mutants with increased p53 activity [9,10,11,12,13], which provided additional evidence of p53 activation in p53^Δ31/Δ31^ mice. However, p53^Δ31/Δ31^ mice exhibited an additional trait not previously reported in this type of mutant: pulmonary fibrosis. In humans, the association of bone marrow failure with pulmonary fibrosis had been proposed to predict germline defects in telomerase [14], which led us to measure telomere lengths in p53^Δ31/Δ31^ cells. Indeed, p53^Δ31/Δ31^ bone marrow cells exhibited a two-fold decrease in average telomere length compared with WT cells, and p53^Δ31/Δ31^ MEFs were also shown to exhibit short and dysfunctional telomeres [6]. Thus, p53^Δ31/Δ31^ mice recapitulated the pathological features (bone marrow failure, pulmonary fibrosis) and the molecular diagnostic feature (short telomeres) of dyskeratosis congenita (DC), the archetypal syndrome of defective telomere maintenance, caused by mutations in genes encoding subunits of the telomerase or shelterin complexes or telomerase regulators [15]. Interestingly, before DC was recognized as a telomere biology disorder, patients were diagnosed with DC when presenting two out of three physical traits: skin hyperpigmentation, nail dystrophy, and oral leukoplakia [16]. All p53^Δ31/Δ31^ mice exhibited skin hyperpigmentation and oral leukoplakia, whereas nail dystrophy was observable but rare. Furthermore, short stature, heart hypertrophy, testicular atrophy, and cerebellar hypoplasia are features observed in patients with Hoyeraal–Hreidarsson syndrome (HHS), a severe variant of DC, and 30% of the p53^Δ31/Δ31^ mice had a hypoplastic cerebellum, 63% a short stature, and 100% a hypertrophic heart, and 91% of male mice exhibited testicular hypoplasia. Interestingly, 10% of the heterozygous p53^+/Δ31^ mutant mice died within a year with a hypertrophic heart, suggesting that they might be mildly affected. Consistent with this, when we analyzed heterozygous p53^+/Δ31^ mutants in a genetic context hemizygous for the p53 negative regulator Mdm4, 70% of the p53^+/Δ31^ Mdm4^+/−^ mice died in less than 3 months, exhibiting bone marrow hypocellularity and short telomeres [6].

Together, these data revealed that a germline increase in p53 activity could cause defects in telomere maintenance in mice, a rather paradoxical finding given the well-accepted notion of “p53, guardian of the genome” [17]. In fact, subtelomeric p53 was later proposed to protect telomeres by stimulating TERRA transcription, in apparent contradiction with p53^Δ31/Δ31^ data [18], suggesting that p53 might act both as a positive and a negative regulator of telomere function [19]. The fact that p53^Δ31/Δ31^ mice recapitulated all the phenotypic traits of DC was also surprising, given the previous phenotypes of mouse models of DC. Indeed, laboratory mice have much longer telomeres than humans, and mice lacking telomerase were reported to exhibit short telomeres only after three or four generations of intracrosses [20,21], whereas short telomeres were observed in the first generation of p53^Δ31/Δ31^ mice [6]. In that respect, p53^Δ31/Δ31^ mice more closely resembled mice with a telomerase haploinsufficiency and a deficient shelterin complex, reported to develop some DC features in one generation—although neither oral leukoplakia nor pulmonary fibrosis [22,23]. This suggested that p53^Δ31^ might exert pleiotropic effects on telomeres, possibly by impacting both telomerase and shelterin complexes. We thus analyzed if genes mutated in dyskeratosis congenita or implicated in aplastic anemia might be regulated by p53. Ten genes corresponding to these criteria were tested, and four were found downregulated by p53: *Dkc1*, *Rtel1*, *Tinf2*, and *Terf1* [6]. Interestingly, the p53^Δ31/Δ31^ mice in our study were of mixed genetic backgrounds (C57Bl/6J and 129S2/SvPas), which made it possible to correlate decreased *Rtel1* gene expression with decreased p53^Δ31/Δ31^ mouse survival, further indicating that an abnormal p53-mediated regulation of *Rtel1* might have pathological consequences [6]. Importantly, p53 activation also led to decreased *DKC1* and *RTEL1* expression in human cells. Furthermore, the phenotypes resulting from additional nonsense *Trp53* mutations targeted in mouse cells, when compared with the distribution of *TP53* nonsense mutations in human cancers, suggested that p53 nonsense mutations might have similar consequences in humans and mice [6]. Together, these data suggested that our observations in mice might be relevant to human disease processes.

## 2. The Plot Thickens: Germline p53 Activation Underlies Features of Several Bone Marrow Failure Syndromes

p53^Δ31/Δ31^ mice phenocopied syndromes of defective telomere maintenance in a single mouse generation, which suggested that p53 had pleiotropic effects on telomeres, and consistent with this, p53 was found to downregulate three genes mutated in dyskeratosis congenita and one gene implicated in aplastic anemia. However, we initially tested only 10 genes associated with dyskeratosis congenita or aplastic anemia, but several tens of proteins were known to impact telomere maintenance [24] and 30–40% of patients with dyskeratosis congenita remained unexplained at the molecular level [25]. This suggested that p53 might regulate additional genes important for telomere maintenance, leading us to test the potential regulation by p53 of 42 other genes in a follow-up study [26]. The 42 tested candidates included genes recently found mutated in syndromes of defective telomere maintenance (e.g., *Tpp1*, *Parn*) [27,28], genes mutated in diseases not primarily associated with telomere biology but for which some patients were shown to exhibit dysfunctional telomeres (e.g., *Fancd2*, *Recql4*) [29,30], genes encoding subunits of the telomerase or shelterin complexes (e.g., *Gar1*, *Terf2*) [24], or other proteins contributing to telomere replication or maintenance. Out of 42 tested genes, 7 were found to be downregulated by p53: *Blm*, *Dek*, *Fancd2*, *Fen1*, *Gar1*, *Recql4*, and *Timeless* [26]. Because *Gar1* encodes a subunit of the telomerase complex, the finding that it is downregulated by p53 provided further evidence that p53 regulates telomere maintenance. However, the gene most dramatically downregulated upon p53 activation was *Fancd2*. This was unexpected, because patients with a *FANCD2* mutation are diagnosed with Fanconi anemia (FA), a bone marrow failure syndrome caused by defective DNA repair, rather than telomere dysfunction. Intriguingly, *Blm* encodes a helicase associated with Fanconi proteins in a multienzyme complex [31], *Fen1* encodes an endonuclease stimulated by Fanca [32], and *Rtel1* encodes a Fancj-like helicase [33], which led us to further investigate the link between p53 and the FA DNA repair pathway. We found that murine p53 downregulates 12 genes in the FA DNA repair pathway, encoding proteins from the FA core complex and its accessory protein (*Fanca*, *Fancb*, *Fancm*, *Ube2t*), the pivotal ID2 complex (*Fancd2*, *Fanci*), and downstream effector proteins (*Brca1*, *Brca2*, *Brip1*, *Palb2*, *Rad51*, *Rad51c*) [26]. Accordingly, p53^Δ31/Δ31^ cells exhibited a typical feature of cells from FA patients: the decreased capacity to repair DNA interstrand crosslinks induced by mitomycin C. Importantly, out of the 12 FA genes downregulated by p53 in mouse cells, 9 were also downregulated by p53 in human fibroblasts, and an increased expression of FA genes could be used as a marker of human tumor progression [26]. Furthermore, HCT116 cells (human colon carcinoma cells expressing a WT p53) were sensitized to mitomycin C upon p53 activation [26], and p53 activation improved platinum-based anti-cancer chemotherapy in a preclinical model [34]. Thus, again, our observations in mice appeared relevant to human disease processes. In sum, p53^Δ31/Δ31^ mice exhibited all the typical features of DC, but their cells also phenocopied cells from patients with FA [6,26].

The phenotypes associated with human mutations causing germline p53 activation also led to a more complex picture. Following the description of p53^Δ31/Δ31^ mice, Tummala et al. reported a mutation in *PARN*, encoding a polyadenylate-specific ribonuclease, in three families with severe DC [27]. This appeared consistent with the p53^Δ31^ mouse model, because PARN was proposed to regulate p53 mRNA stability [35] and because PARN-depleted cells exhibited an altered p53-dependent response to DNA damage and decreased mRNA levels for *DKC1*, *RTEL1*, and *TERF1* [27], three genes also downregulated in p53^Δ31/Δ31^ mice [6]. However, PARN also regulates TERC, the telomerase RNA component [36]. Accordingly, decreased TERC levels were also found in PARN-depleted cells [27], and TERC overexpression increased telomere length in PARN-deficient cells [37], raising the possibility that the effect of PARN on telomeres might primarily result from its capacity to regulate TERC, rather than p53.

Toki et al. next reported de novo germline *TP53* mutations in two patients with an atypical bone marrow failure syndrome [38]. In both patients, heterozygous frameshift *TP53* mutations were found which both led to a hyperactive mutant p53 protein truncated of its 32 C-terminal residues (p53^Δ32^). Both patients exhibited pure red cell aplasia, growth delays, hypogammaglobulinemia, microcephaly, and telomere length between the 1st and 10th percentile, and one patient also presented reticular skin pigmentation, hypogonadism, and tooth anomalies. These phenotypes were suggestive of a DBA-like syndrome with additional features more common in DC [38]. Additional patients with similar *TP53* mutations were later confirmed to present DBA-like features, but could exhibit normal telomere length [39,40].

In collaboration with Sharon Savage, we identified a family with a heterozygous germline missense mutation in *MDM4.* In this family, carriers of the *MDM4^T454M^* mutation exhibited considerable heterogeneity in their phenotypes, with features suggestive of DC, e.g., bone marrow hypocellularity, short telomeres, tongue squamous cell carcinoma, and acute myeloid leukemia [41]. We created a mouse model with the same *Mdm4* missense mutation (p.T454M) and found that Mdm4^T454M/T454M^ MEFs exhibited decreased Mdm4 protein levels, increased p53 activity, and short telomeres. Mdm4^T454M/T454M^ mice were born in Mendelian proportions but were smaller than their WT and Mdm4^+/T454M^ littermates, and they died immediately after birth from neonatal respiratory failure. Importantly however, 80% of the Mdm4^T454M/T454M^ p53^+/−^ mice died from bone marrow failure in 2–6 months, which indicated that the Mdm4^T454M^ mutant is extremely sensitive to a decrease in p53 signaling. Conversely, most Mdm4^+/T454M^ heterozygous mice remained alive after 6 months and appeared similar to WT mice, except for a very subtle increase in skin pigmentation in 30% of animals. By contrast, all the Mdm4^+/T454M^ p53^+/Δ31^ compound heterozygotes died in less than 3 months and exhibited intense skin hyperpigmentation, short stature, cardiac hypertrophy, testicular hypoplasia, bone marrow failure, and short telomeres, again indicating that the Mdm4^T454M^ mutant is extremely sensitive to variations in p53 signaling [41]. These observations in mice suggested that variations in p53 activity might account for the variable expressivity and penetrance of clinical features among the MDM4^+/T454M^ human family relatives. Consistent with this, the most affected family member, with a lymphocyte telomere length within or below the first percentile of age-matched participants and a tongue squamous cell carcinoma at age 27, exhibited a WT *MDM4* allele with three single-nucleotide polymorphisms (SNPs) associated with increased p53 activity in addition to the *MDM4^T454M^* allele. Opposite this, less severely affected family members presented SNPs in *MDM4* or *TP53* that might antagonize the effects of the MDM4^T454M^ mutation [41]. Importantly, five independent patients with a germline mutation in *MDM4* were reported in two additional studies: all presented bone marrow abnormalities, and two exhibited very short telomeres, thus confirming the impact of MDM4 on telomere maintenance [42,43].

Germline mutations of MDM2, another p53 negative regulator, were also reported. A homozygous germline antiterminating mutation in *MDM2* was found in a patient affected by a Werner-like segmental progeroid syndrome, with clinical traits notably including short stature, prematurely gray hair, and testicular hypoplasia [44]. Although telomere attrition is a primary hallmark of aging known to trigger cellular senescence [45], the potential link between p53 activation and telomere attrition was not investigated in this report. In another study, genomic DNA from 179 patients with bone marrow failure of suspected inherited origin was sequenced, and likely pathogenic variants in *TP53*, *MDM4*, or *MDM2* were found in a few patients, but were not shown to cause disease phenotypes [46]. Thus, whether a germline mutation in *MDM2* may cause bone marrow failure syndromes remains uncertain at this time.

Altogether, the studies in mice and humans indicate that mutations leading to a germline activation of the p53 pathway may cause bone marrow failure, with variable phenotypic features that resemble DC, DBA, or FA.

## 3. Food for Thought: Modeling p53 in a Circuitry of Genes Associated with Bone Marrow Failure Syndromes

Genes whose mutations cause DC, FA, or DBA will lead to distinct molecular outcomes, i.e., defective telomeres, unrepaired DNA interstrand crosslinks, or dysfunctional ribosomes, respectively [15]. Importantly however, these distinct molecular outcomes have the same consequence: they all lead to p53 activation [9,47,48,49,50,51,52], which may also result from specific germline mutations in the *Trp53*/*TP53* [6,38,40] or *MDM4* genes [41,42,43], as reviewed above. In DC, FA, and DBA, p53 activation is known to cause bone marrow failure [49,53,54,55], notably by impairing proliferation or by promoting the apoptosis, differentiation or aging of hematopoietic stem and progenitor cells (HSPCs) [49,54,56,57,58,59]. Notably, however, p53 might also play a compensatory role in preventing the replicative exhaustion of HSPCs in some cases [58]. Interestingly, we found that p53 activation leads to the repression of several genes mutated in DC or FA [6,26], which raised the possibility that the repression of these genes might contribute to bone marrow hypoplasia and disease phenotypes. Consistent with this, we observed a direct correlation between increased p53 activity, decreased *Rtel1* expression, and increased risk of bone marrow failure in p53^Δ31/Δ31^ mice [6]. Furthermore, p53^Δ31/Δ31^ cells exhibited a decreased capacity to repair DNA interstrand crosslinks, a distinctive feature best explained by their increased repression of multiple genes in the Fanconi anemia DNA repair pathway [26]. Interestingly, although DC and FA are caused by mutations in different sets of genes, patients with either syndrome may present similar features (aplastic anemia, abnormal skin pigmentation, short stature, cerebellar or testicular hypoplasia), which may lead to misdiagnosis [60,61]. In fact, telomere dysfunction was reported in some patients with FA [30,62,63], and a hypersensitivity to mitomycin C was also reported for cells from some DC patients [64,65]. Thus, defects in telomere maintenance or in the FA DNA repair pathway were long known to cause p53 activation, but our results indicated that, conversely, increased p53 activity might affect telomere function and DNA repair, hence defining positive feedback loops that might contribute to the clinical overlap between DC and FA [26]. Interestingly, the clinical traits of humans with a germline activating *TP53* mutation also revealed features of DBA [38,39,40], a bone marrow failure syndrome also partially similar to DC or FA, and mutations in DBA-causal genes are also known to cause p53 activation [9,66,67,68,69,70]. In that case, however, evidence for a positive feedback loop was not established, because no DBA-causal gene had been reported to be downregulated by p53. Nevertheless, p53 appeared likely to impact ribosome function, notably by repressing the expression of *Fbl*, encoding a rRNA methyl-transferase [71]. Together, these data suggested that an extensive knowledge of the repertoire of genes downregulated by p53 might provide clues on the molecular mechanisms underlying bone marrow failure. To this goal, we recently designed a systematic approach to identify genes repressed upon p53 activation.

p53 was shown to repress transcription over 30 years ago [72,73], and multiple mechanisms were proposed to account for p53-dependent transcriptional repression over the years [74]. Evidence that p53 may repress a few genes directly by binding to their promoter sequences was provided for a few genes, and *Dkc1* and *Fbl* are among those genes [6,71]. However, most genes downregulated by p53 appear to be repressed indirectly, through the binding to their promoters of a repressor complex called DREAM (dimerization partner, RB-like, E2F4/5, and MuvB) [75,76,77,78,79,80,81,82,83]. Accordingly, we obtained evidence that, upon p53 activation, the binding of the E2F4 repressor (a subunit of DREAM) was increased at the promoters of *Rtel1* and 12 genes of the FA DNA repair pathway, and identified functionally DREAM-binding sites for 3 FA genes (*Fancd2*, *Fanci*, *Rad51*) [26] as well as 2 other p53-regulated genes (*Cenpa*, *Hjurp*) in a later study [84]. Thus, searching for DREAM targets appeared as the most promising approach to identify additional genes repressed by p53.

To search for genes downregulated upon p53 activation, we exploited transcriptomic changes associated with the in vitro differentiation of bone marrow cells (BMCs). The system we analyzed relies on the conditional expression of Hoxa9 [85], a transcription factor required for hematopoiesis [86,87]. In cells overexpressing Hoxa9-ER, tamoxifen withdrawal led to BMC differentiation which, based on the induction of 17 well-known p53-transactivated genes and the downregulation of 7 p53-repressed genes, correlated with p53 activation [88]. To identify p53-DREAM candidate targets relevant to bone marrow failure syndromes, we focused on 3631 genes downregulated at least 1.5-fold upon BMC differentiation and associated with a Gene Ontology (GO) term. Importantly, we first found that genes with GO terms relative to telomere biology, FA DNA repair, or ribosome function were significantly over-represented among these candidates. A total of 571 genes associated with blood-related GO terms and downregulated upon BMC differentiation were identified [88], of which 499 were previously reported to be downregulated upon p53 activation in mouse and/or human cells [89], suggesting the relevance of our approach. E2F4 and LIN9 (two subunits of the DREAM complex) strongly bound to the promoters of 269 of these genes according to public ChIP-seq data [90]. We next analyzed RNAseq data from unirradiated and irradiated hematopoietic stem cells or splenic cells from mice with different p53 statuses [91,92], leading to a list of 213 blood-related genes that appeared as the most relevant candidate p53-DREAM targets. Furthermore, as mentioned above, mice and humans with bone marrow failure syndromes may present microcephaly or cerebellar hypoplasia, which led us to use a similar approach to identify p53-DREAM targets that might contribute to these brain-related abnormalities. A total of 478 genes associated with brain-related GO terms were downregulated at least 1.5-fold upon BMC differentiation [88], of which 408 were reported to be downregulated upon p53 activation in mouse and/or human cells [89], and E2F4 and LIN9 strongly bound to the promoters of 226 of these genes. To estimate the relevance of these 226 genes, we next analyzed RNAseq data from cortical neural cell progenitors infected or not by the Zika virus, a virus known to cause p53 activation in these cells and microcephaly [93,94]. This led to a list of 162 brain-related genes that appeared as the most relevant candidate p53-DREAM targets. Importantly, out of the 162 brain-related candidates identified, 58 also belonged to the list of 213 genes associated with abnormal hematopoiesis, consistent with the notion that a deregulation of the p53-DREAM pathway might be involved in both pathological processes. In sum, this systematic approach suggested 317 genes (213 + 162 − 58) associated with blood or brain abnormalities as potential p53-DREAM targets. We next used successive iterations of positional frequency matrices to identify evolutionary conserved DREAM binding sites in the promoters of these candidate genes. Putative DREAM binding sites were identified for 151 genes, all within regions bound by E2F4 and LIN9 and co-mapping with ChIP peaks in most cases, and 21 of these sites were tested in luciferase assays and shown to alter gene expression [88]. Notably, only a fraction of these genes were previously proposed to be DREAM targets in previous studies [81,89,95], and with apparently less accurate DREAM site predictions [88].

**Figure 1:**
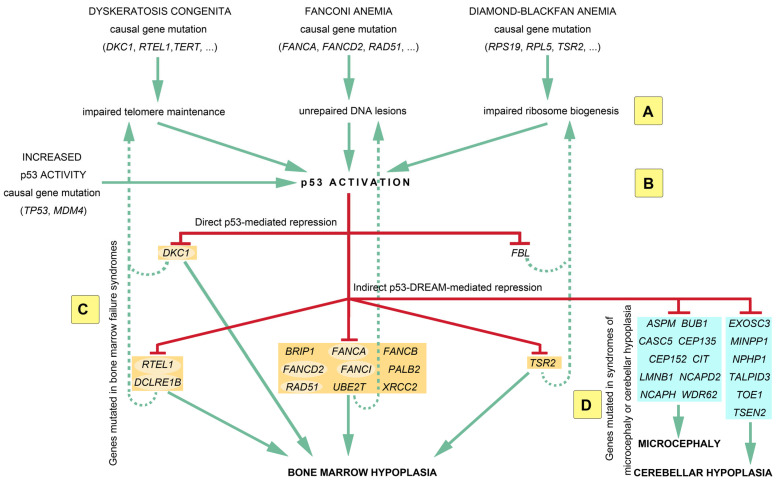
A p53-centric molecular circuitry of genes implicated in bone marrow failure syndromes. Mutations in the genes causing dyskeratosis congenita, Fanconi anemia, or Diamond–Blackfan anemia will lead to distinct molecular outcomes, i.e., defective telomeres, unrepaired DNA interstrand crosslinks, or dysfunctional ribosomes, respectively (**A**). However, these distinct molecular outcomes have the same consequence: they all lead to p53 activation (**B**), which may also result from some germline mutations in the *TP53* or *MDM4* genes. p53 activation leads to the repression of many genes mutated in bone marrow failure syndromes (BMFSs) (orange boxes), which likely contributes to bone marrow hypoplasia (**C**). The p53-mediated repression of BMFS-causal genes might reinforce the initial molecular defect (dashed lines) to cause further p53 activation, defining positive regulatory feedback loops. Furthermore, the large repertoire of p53-repressed genes may contribute to partial clinical overlaps between the different BMFSs. Notably, some p53-repressed genes (highlighted with pale oval backgrounds) have multiple functions, which may also contribute to overlapping phenotypes. Importantly, microcephaly or cerebellar hypoplasia are observed in a fraction of patients with a BMFS, and the p53-DREAM pathway also represses (**D**) several genes mutated in these brain disorders (blue boxes). See text for additional details.

DREAM binding sites were notably identified for genes mutated in dyskeratosis congenita (*RTEL1*, *DCLRE1B*), Fanconi anemia (*FANCA*, *FANCB*, *FANCD2*, *FANCI*, *BRIP1*, *PALB2*, *RAD51*, *UBE2T*, *XRCC2*), and Diamond–Blackfan anemia (*TSR2*). These results suggest a model of molecular circuitry that may account for the partial clinical overlap between bone marrow failure syndromes of distinct molecular origins: independently of the initial causal mutation, the resulting molecular defect will lead to p53 activation, and this will lead to the repression of a set of genes implicated in telomere maintenance, DNA repair, and ribosome function (Figure 1). Due to positive feedback loops, the repression of these genes may lead to further p53 activation. Notably, some p53-repressed genes apparently have multiple functions, which may also contribute to overlapping clinical features. For example *DKC1, DCLRE1B*, and *RTEL1* are crucial for telomere maintenance [96], but *DKC1* was also implicated in ribosomal function [97], whereas *DCLRE1B* and *RTEL1* were also implicated in DNA repair [98,99]. Likewise, *FANCA*, *FANCD2*, *FANCI*, and *RAD51* are crucial for DNA repair [100], but *FANCI* may also impact ribosomal function [101], *FANCD2* or *RAD51* may play a role in telomere maintenance [30,102], and *FANCA* may act on both telomere maintenance and ribosomal function [103,104]. Importantly, p53 activation also leads to the DREAM-mediated repression of genes associated with syndromes of microcephaly (*ASPM*, *BUB1*, *CASC5*, *CEP135*, *CEP152*, *CIT*, *LMNB1*, *NCAPD2*, *NCAPH*, *WDR62*) or cerebellar hypoplasia (*EXOSC3*, *MINPP1*, *NPHP1*, *TALPID3*, *TOE1*, *TSEN2*), which might account for the brain-related phenotypes often observed in patients with DC [105], FA [100], and DBA [106] (Figure 1).

**Figure 2:**
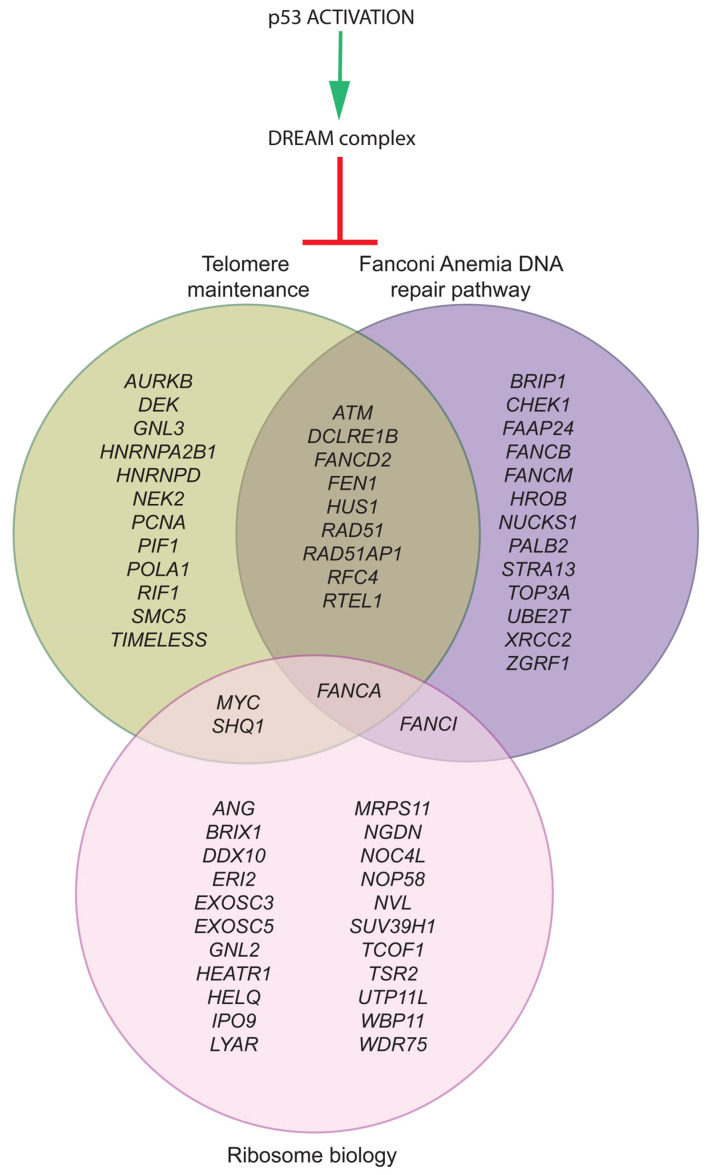
The p53-DREAM pathway represses 60 genes relevant to DC, FA, and DBA. Out of the 151 p53-DREAM targets we identified, 60 were associated with gene ontology terms (in mice and humans) and bibliographic data for telomere maintenance (green), DNA interstrand crosslink repair (purple), or ribosome biology (pink). The classification of p53-DREAM targets shown here relies on 42 relevant GO terms (for details, see Ref. [88]) and additional bibliographic data [30,32,97,98,99,101,102,103,104,109,110,111,112,113,114,115,116].

It should be noted that the molecular circuitry represented in Figure 1 includes only 28 of the 151 p53-DREAM targets identified and is, therefore, a simplified view. Figure 2 provides a more complete view of p53-DREAM target genes related to DC, FA, and DBA, but not necessarily mutated in either of these syndromes. In this Figure, sixty blood-related p53-DREAM targets are shown. They correspond to genes downregulated by p53, associated with GO terms relative to telomere maintenance, DNA interstrand crosslink repair or ribosome biology, and with promoter sequences bound by DREAM and containing an evolutionary conserved and appropriately mapped DREAM-binding site. Notably, GO term lists for murine genes were used in ref. [88], but we performed here a more comprehensive analysis by relying on GO term lists for both mice and humans and by integrating additional bibliographic data. Importantly, 13 out of the 60 genes are currently known to be implicated in two or three biological functions, which provides further evidence that these functions are intertwined (Figure 2). Furthermore, we should emphasize that with our current method, putative DREAM-binding sites were identified in the promoters of about half of the candidate p53-DREAM targets. Most notably, the FA-causal genes *BRCA1* and *BRCA2* appear as very good candidate p53-DREAM targets [26,88] for which DREAM-binding sites remain to be identified. In addition, some p53-DREAM target genes, the mutation of which may cause hematopoietic defects, were not included in Figure 2 because they are not associated with telomere, Fanconi, or ribosome-related GO terms. This is notably the case for *NUF2* and *MTHFD1* [107,108]. Thus, the impact of p53 on hematopoiesis includes additional genes that are not represented in Figure 2.

## 4. Conclusions

Ten years after finding that mice with a germline activating p53 mutation exhibit features of a telomere disorder syndrome, we now know that p53 downregulates the expression of many genes implicated in telomere maintenance, DNA repair, and ribosome biology. This may contribute to explaining why p53 activation plays a central role in bone marrow failure processes, and account for the partial clinical overlap between inherited bone marrow failure syndromes. Interestingly, these studies revealed positive feedback loops that may vary in strength because of SNPs affecting the *TP53* or *MDM4* genes, which might account for the variable expressivity of disease symptoms among family members with the same syndrome-causing mutation. Evidence for this was already obtained in a familial syndrome of defective telomere maintenance caused by a *MDM4* mutation [41], and it will be important to determine if this may also apply to other bone marrow failure syndromes caused by mutation in other genes. Furthermore, because p53 is a tumor suppressor whose activity is frequently lost in cancer cells, the p53-DREAM target genes identified may be overexpressed upon p53 inactivation, and thus behave as markers of tumor progression [26] or even promote tumorigenesis. In the future, strategies to tweak the p53-DREAM pathway might thus be useful to treat patients with either bone marrow failure or cancer.

## Data Availability

No new data were created or analyzed in this study. Data sharing is not applicable to this article.
